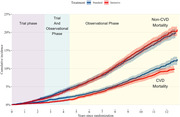# Long‐term mortality in the SPRINT cohort

**DOI:** 10.1002/alz.095416

**Published:** 2025-01-09

**Authors:** Byron C Jaeger, Sarah A. Gaussoin, Jeff D. Williamson, Nicholas M. Pajewski, David M. Reboussin

**Affiliations:** ^1^ Wake Forest University School of Medicine, Winston‐Salem, NC USA

## Abstract

**Background:**

The Systolic Blood Pressure Intervention Trial (SPRINT) showed intensive blood pressure (BP) control, defined by systolic BP goal of < 120 mm Hg, reduced cardiovascular morbidity and mortality. However, a secondary analysis that incorporated approximately 4‐years of observational follow‐up found that mean BP levels increased after the trial and the benefit of intensive BP control on cardiovascular and all‐cause mortality attenuated. We re‐examined these findings using an updated version of the observational data that includes approximately 8‐years of follow‐up.

**Methods:**

SPRINT included patients 50 years and older with hypertension and increased cardiovascular risk but without diabetes or history of stroke. The SPRINT intervention ended on August 20, 2015, and trial close‐out visits occurred through July 2016. Extended observational follow‐up for mortality was obtained via the US National Death Index from 2016 through 2023.

**Results:**

Among 9361 randomized participants, the mean (standard deviation) age was 67.9 (9.4) years, and 3332 (35.6%) were women. Over a maximum follow‐up of 13 years, 2597 all‐cause and 907 CVD mortality events occurred (**Figure**). During the trial, intensive treatment was beneficial for both cardiovascular mortality (hazard ratio [HR], 0.66; 95% CI, 0.49‐0.89) and all‐cause mortality (HR, 0.83; 95% CI, 0.68‐1.00). During the 8‐year observational period, the benefit of intensive BP control attenuated for both outcomes (CVD mortality HR: 1.02; 95% CI, 0.84‐1.24; all‐cause mortality HR: 1.08; 95% CI, 0.94‐1.23). Overall (i.e., combining trial and observational periods), intensive BP control was beneficial for CVD mortality (HR, 0.84; 95% CI, 0.74‐0.96) but not all‐cause mortality (HR, 0.96; 95% CI, 0.89‐1.04).

**Conclusions:**

Intensive BP control reduced CVD mortality risk during the trial and overall, but its benefits were less evident during the observational period. Given increasing BP levels during the observational period, maintaining BP control may be essential for sustaining long‐term benefit.

Figure: Cumulative incidence of cardiovascular and non‐cardiovascular mortality by treatment group